# Supplementary material for: Conjunctival Microbiome-Host Responses Are Associated With Impaired Epithelial Cell Health in Both Early and Late Stages of Trachoma
Source: Front Cell Infect Microbiol. 2019 Aug 21;9:297. doi: 10.3389/fcimb.2019.00297 (PMC6736612; doi:10.3389/fcimb.2019.00297)
Supplement: Supplementary file 1 [file Data_Sheet_1.pdf]

Conjunctival microbiome-host responses are associated with impaired epithelial cell health in both early and late stages of trachoma

Supplementary Tables

Supplementary Table 1. TLDA card details

General category*	Target
Anti-microbial peptides[1-4]	DEFB4B_DEFB4A, S100A7,
Cell adhesion/growth regulation[1]	CEACAM1, CD53
Cell cycle/proliferation[1]	CDK13, PRDX3, TP53
Cell markers[1]	CD247, KLRF1
Cytokines/chemokines[1, 2, 4]	CCL2, IFNG, IL12B, IL17A, IL22, IL23A, TNFSF15
Mucins/matrix modifiers[2, 4, 5]	MMP7, MUC1, MUC4, MUC5AC, MUC7, MUC16, SPARCL1
Pattern recognition/inflammasome[1]	NLRP1, NLRP3, NLRP6, NOD1, NOD2, RIPK2, TLR4, TLR6
Regulators/signaling pathways[1]	CHD8, COMMD6, MAPK14, MYD88, NFKB1, PDL1, SRGN, TRAF6
Regulatory elements[6, 7]	miR-147B, miR-155, miR-184, miR-1285
Response to microbiota[1, 4]	ALOX5AP, ANAX1, BCL2, CD40, REL, TNFRSF1A, TNFRSF1B

Reference for evidence of differential regulation in trachoma

Supplementary Table 2. Gene expression sample demographics

Phenotype	Children (< 16 years of age)			Adults (>= 16 years of age)		
	Healthy n = 32 (41%)	Active trachoma n = 46 (59%)	Adjusted p-value	Healthy n = 69 (47%)	Scarring trachoma n = 78 (53%)	Adjusted p-value
**Male (%)**	18 (56)	29 (39)	0.713	22 (32)	16 (21)	0.167
**Median age (range)**	6 (1-13)	5 (1-13)	0.291	47 (16-87)	50 (16-84)	0.063
**Sample collection [n(%]**			0.884			1.000
dry season	5 (16)	9 (20)		51 (74)	57 (73)	
wet season	27 (84)	37 (80)		18 (26)	21 (27)	
**Ethnicity [n(%)]**			0.439			0.495
Jola	5 (16)	6 (13)		23 (33)	23 (29)	
Mandinka	14 (44)	17 (37)		30 (43)	43 (55)	
Wolof	10 (31)	12 (26)		6 (9)	4 (5)	
Other	3 (9)	11 (24)		10 (14)	8 (10)	
**Administrative district [n(%)]**			0.170			0.456
Basse	0 (0)	0 (0)		7 (10)	9 (12)	
Brikama	10 (31)	15 (33)		34 (49)	39 (50)	
Janjanbureh	1 (3)	0 (0)		1 (1)	0 (0)	
Kanifing	0 (0)	0 (0)		1 (1)	1 (1)	
Kerewan	0 (0)	3 (7)		6 (9)	4 (5)	
Kuntaut	2 (6)	0 (0)		3 (4)	4 (5)	
Mansa Konko	19 (59)	28 (61)		8 (12)	21 (27)	
Unknown	0 (0)	0 (0)		9 (13)	0 (0)	

Supplementary Table 3. Univariate and modular gene expression in children

**Module** *putative function*	**Target**	**Fold-change***	**Adjusted p-value**
ATEM1** *cellular adhesion and division*	ANXA1	1.148	0.636
	BCL2	1.146	0.689
	CD247	1.212	0.520
	CEACAM1	1.326	0.810
	IFNG	1.174	0.260
	IL12B	0.507	0.104
	IL22	0.498	0.004
	KLRF1	1.930	0.355

	MMP7	1.090	0.881
	miR-1285	0.494	0.372
	miR-147B	1.010	0.881
	miR-155	1.300	0.101
	miR-184	0.934	0.668
ATEM2<br>*innate response to microbiota*	ALOX5AP	0.871	0.288
	CCL2	0.901	0.347
	CD274	0.971	0.756
	CD53	0.664	0.017
	COMMD6	0.559	0.101
	DEFB4B_DEFB4A	1.122	0.347
	IL17A	0.890	0.421
	IL23A	1.349	0.101
	MUC7	0.952	0.756
	NLRP3	0.797	0.143
	NLRP6	1.066	0.689
	NOD2	1.028	0.843
	RIPK2	0.935	0.602
	S100A7	0.907	0.602
	SRGN	0.709	0.003
	TLR4	1.300	0.142
	TLR6	1.849	0.118
	TNFRSF1B	1.520	0.003
	TNFSF15	1.096	0.520
ATEM3<br>*suppression of epithelial cell expansion/recovery*	CD40	1.132	0.756
	CDK13	1.290	0.243
	CHD8	1.602	0.041
	MAPK14	1.395	0.101
	MUC1	1.416	0.055
	MUC16	1.323	0.229
	MUC4	0.950	0.881
	MUC5AC	1.021	0.881
	MYD88	2.077	0.003
	NFKB1	1.139	0.494
	NLRP1	2.921	0.024
	NOD1	2.587	0.003
	PRDX3	2.199	0.006
	REL	1.569	0.143
	SPARCL1	1.047	0.750
	TNFRSF1A	1.324	0.103
	TP53	3.919	0.005
	TRAF6	0.198	0.003

**Fold-change in expression from healthy controls to active trachoma cases*
***ATEM – active trachoma expression module*

Supplementary Table 4. Univariate and modular gene expression in adults

**Module**<br>*putative function*	**Target**	**Fold-change***	**Adjusted p-value**
STEM1**<br>*epithelial health*	COMMD6	1.148	0.342
	IFNG	1.146	0.010
	IL12B	1.212	0.495
	IL22	1.326	0.006
	KLRF1	1.174	0.668
	MUC7	0.507	0.077
	TNFSF15	0.498	0.008
	miR-147B	1.930	0.550
	miR-155	1.090	0.275
	miR-184	0.494	0.902
STEM2<br>transcriptional control of innate sensing	BCL2	1.010	0.687
	CD247	1.300	0.146
	CD40	0.934	0.061
	CDK13	0.871	0.797
	CHD8	0.901	0.004

	MAPK14	0.971	0.495
	MUC16	0.664	0.535
	MUC5AC	0.559	0.294
	MYD88	1.122	0.036
	NLRP1	0.890	0.832
	NLRP3	1.349	0.391
	NOD1	0.952	0.239
	PRDX3	0.797	0.130
	REL	1.066	0.130
	RIPK2	1.028	0.294
	TNFRSF1A	0.935	0.888
	TP53	0.907	0.271
	TRAF6	0.709	0.169
STEM3 *cellular proliferation and activation*	ALOX5AP	1.300	0.130
	CCL2	1.849	0.787
	CD53	1.520	0.172
	NFKB1	1.096	0.019
	NLRP6	1.132	0.294
	NOD2	1.290	0.106
	SRGN	1.602	0.124
	TLR4	1.395	0.259
	TLR6	1.416	0.888
	TNFRSF1B	1.323	0.943
	miR-1285	0.950	0.007
STEM4 *response to microbiota*	ANXA1	1.021	0.124
	CD274	2.077	0.275
	CEACAM1	1.139	0.137×10^{6}
	DEFB4B_DEFB4A	2.921	0.003
	IL17A	2.587	0.457
	IL23A	2.199	0.004
	MMP7	1.569	0.004
	MUC1	1.047	0.110×10^{5}
	MUC4	1.324	0.137×10^{7}
	S100A7	3.919	0.463
	SPARCL1	0.198	0.001

**Fold-change in expression from healthy controls to scarring trachoma cases*
***STEM – scarring trachoma expression module*

Supplementary Table 5. 16S sample demographics

Phenotype	Children (< 16 years of age)			Adults (>= 16 years of age)		
	Healthy (31[43%])	Active trachoma (41[57%])	Adjusted p-value	Healthy (105[45%])	Scarring trachoma (130[55%])	Adjusted p-value
**Male (%)**	18 (58)	26 (63)	0.828	31 (24)	34 (26)	0.669
**Median age (range)**	5 (1-14)	5 (1-12)	0.382	52 (16-87)	53 (16-84)	0.188
**Sample collection [n(%]**			1.000			1.000
dry season	4 (13)	6 (15)		78 (74)	97 (75)	
wet season	27 (87)	35 (85)		27 (26)	33 (25)	

**Ethnicity [n(%)]**			0.705			0.763
Jola	6 (19)	6 (15)		36 (34)	44 (34)	
Mandinka	12 (39)	13 (32)		43 (41)	59 (45)	
Wolof	8 (26)	11 (27)		9 (9)	7 (5)	
Other	5 (16)	11 (27)		17 (16)	20 (15)	
**Administrative district [n(%)]**			0.530			0.917
Basse	0 (0)	0 (0)		8 (8)	12 (9)	
Brikama	11 (35)	13 (32)		57 (54)	71 (55)	
Janjanbureh	0 (0)	0 (0)		2 (2)	3 (2)	
Kanifing	0 (0)	0 (0)		1 (1)	1 (1)	
Kerewan	0 (0)	1 (2)		8 (8)	10 (8)	
Kuntaut	1 (3)	0 (0)		5 (5)	5 (4)	
Mansa Konko	19 (61)	27 (66)		14 (13)	28 (22)	
Unknown	0 (0)	0 (0)		10 (9)	0 (0)	

Supplementary Table 6. Host genotype in adults by case-control status

	**Adults (>= 16 years of age)**		
**Phenotype**	Healthy (121[43%])	Scarring trachoma (158[57%])	Adjusted p-value
**KIR2DL2 copy number [n(%)]**			0.412
0	45 (37)	71 (45)	
1	33 (27)	45 (28)	
2	6 (5)	4 (3)	
Unknown	37 (31)	38 (24)	
**KIR2DL3 copy number [n(%)]**			0.492
0	14 (12)	14 (9)	
1	47 (39)	77 (49)	
2	22 (18)	31 (20)	
Unknown	38 (31)	36 (23)	
**HLA-C genotype [n(%)]**			0.228
C1	27 (22)	47 (30)	
C1C2	50 (41)	64 (41)	
C2	26 (22)	24 (15)	
Unknown	18 (15)	23 (15)	
**NKG2C genotype [n(%)]**			0.605
WT/WT	47 (39)	53 (34)	
WT/DEL	55 (45)	77 (49)	
DEL/DEL	16 (13)	25 (16)	
Unknown	3 (3)	3 (2)	

Supplementary Figures

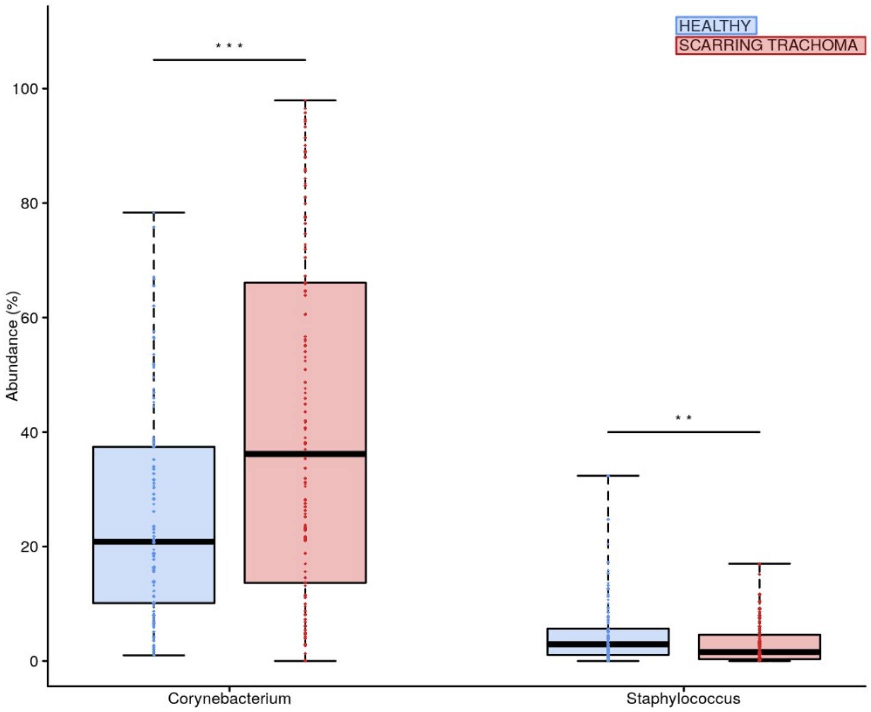

Supplementary Figure 1. Univariate analysis of relative abundance of genera by case-control status in adults.

Genera with significantly different relative abundance between scarring trachoma cases (red) and healthy controls (blue) are shown. Boxes represent the interquartile range, with median indicated (blue line). Outer bars represent the range. P-values were considered significant at <0.05 and are denominated as follows: * p<0.05; ** p<0.01; *** p<0.001.

References

1. Natividad A, Freeman TC, Jeffries D, Burton MJ, Mabey DC, Bailey RL, et al. Human conjunctival transcriptome analysis reveals the prominence of innate defense in Chlamydia trachomatis infection. Infect Immun. 2010 Nov;78(11):4895-911
2. Burton MJ, Rajak SN, Bauer J, Weiss HA, Tolbert SB, Shoo A, et al. Conjunctival transcriptome in scarring trachoma. Infect Immun. 2011 Jan;79(1):499-511
3. Burton MJ, Rajak SN, Hu VH, Ramadhani A, Habtamu E, Massae P, et al. Pathogenesis of progressive scarring trachoma in Ethiopia and Tanzania and its implications for disease control: two cohort studies. PLoS Negl Trop Dis. 2015 May;9(5):e0003763
4. Ramadhani AM, Derrick T, Macleod D, Massae P, Mtuy T, Jeffries D, et al. Immunofibrogenic Gene Expression Patterns in Tanzanian Children with Ocular Chlamydia trachomatis Infection, Active Trachoma and Scarring: Baseline Results of a 4-Year Longitudinal Study. Front Cell Infect Microbiol. 2017;7:406
5. Holland MJ, Jeffries D, Pattison M, Korr G, Gall A, Joof H, et al. Pathway-focused arrays reveal increased matrix metalloproteinase-7 (matrilysin) transcription in trachomatous trichiasis. Invest Ophthalmol Vis Sci. 2010 Aug;51(8):3893-902
6. Derrick T, Roberts C, Rajasekhar M, Burr SE, Joof H, Makalo P, et al. Conjunctival MicroRNA expression in inflammatory trachomatous scarring. PLoS Negl Trop Dis. 2013;7(3):e2117
7. Derrick T, Last AR, Burr SE, Roberts CH, Nabicassa M, Cassama E, et al. Inverse relationship between microRNA-155 and -184 expression with increasing conjunctival inflammation during ocular Chlamydia trachomatis infection. BMC Infect Dis. 2016 Feb 3;16:60
